# Innate Immunity Components and Cytokines in Gastric Mucosa in Children with *Helicobacter pylori* Infection

**DOI:** 10.1155/2015/176726

**Published:** 2015-04-08

**Authors:** Jacek Michalkiewicz, Anna Helmin-Basa, Renata Grzywa, Mieczyslawa Czerwionka-Szaflarska, Anna Szaflarska-Poplawska, Grazyna Mierzwa, Andrzej Marszalek, Magdalena Bodnar, Magdalena Nowak, Katarzyna Dzierzanowska-Fangrat

**Affiliations:** ^1^Chair of Immunology, Collegium Medicum Nicolaus Copernicus University, M. Sklodowskiej-Curie 9, 85-094 Bydgoszcz, Poland; ^2^Department of Clinical Microbiology and Immunology, The Children's Memorial Health Institute, Aleja Dzieci Polskich 20, 04-730 Warsaw, Poland; ^3^Department of Pediatrics, Allergology and Gastroenterology, Collegium Medicum Nicolaus Copernicus University, M. Sklodowskiej-Curie 9, 85-094 Bydgoszcz, Poland; ^4^Department of Pediatric Endoscopy and Gastrointestinal Function Testing, Collegium Medicum Nicolaus Copernicus University, M. Sklodowskiej-Curie 9, 85-094 Bydgoszcz, Poland; ^5^Department of Clinical Pathomorphology, Collegium Medicum Nicolaus Copernicus University, M. Sklodowskiej-Curie 9, 85-094 Bydgoszcz, Poland; ^6^Department of Oncologic Pathology, The Greater Poland Cancer Centre, Garbary 15, 61-866 Poznan, Poland; ^7^Institute of Nursery and Public Health, Rzeszow University, Al. Rejtana 16A, 35-310 Rzeszow, Poland

## Abstract

*Purpose.* To investigate the expression of innate immunity components and cytokines in the gastric mucosa among *H. pylori* infected and uninfected children. *Materials and Methods.* Biopsies of the antral gastric mucosa from children with dyspeptic symptoms were evaluated. Gene expressions of innate immunity receptors and cytokines were measured by quantitative real-time PCR. The protein expression of selected molecules was tested by immunohistochemistry. *Results. H. pylori* infection did not lead to a significant upregulation of *MyD88, TLR2, TLR4, CD14, TREM1,* and *TREM2* mRNA expression but instead resulted in high mRNA expression of *IL-6, IL-10, IFN-γ, TNF-α,* and *CD163. H. pylori cagA*(+) infection was associated with higher *IL-6* and *IL-10* mRNA expression, as compared to *cagA*(−) strains. *H. pylori* infected children showed increased IFN-*γ* and TNF-*α* protein levels. IFN-*γ* mRNA expression correlated with both *H. pylori* density of colonization and lymphocytic infiltration in the gastric mucosa, whereas TNF-*α* protein expression correlated with bacterial density. *Conclusion. H. pylori* infection in children was characterized by (a) Th1 expression profile, (b) lack of mRNA overexpression of natural immunity receptors, and (c) strong anti-inflammatory activities in the gastric mucosa, possibly resulting from increased activity of anti-inflammatory M2 macrophages. This may explain the mildly inflammatory gastric inflammation often observed among *H. pylori* infected children.

## 1. Introduction

Gastric mucosa epithelial cells and myeloid cells (monocytes, macrophages, and dendritic cells) form the first barrier to* Helicobacter pylori* (*H. pylori*) infection. They recognize bacteria through pattern recognition receptors (PRRs), which interact with conserved microbial structures called pathogen-associated molecular patterns (PAMPs).

One of the PRRs systems involved in* H. pylori* recognition is a family of Toll-like receptors (TLRs). TLRs are present both on gastric epithelial cells and on immune cells infiltrating gastric mucosa. TLRs in the gastric mucosa involve 5 members of this family [1–3]. Studies on epithelial cell lines showed that* H. pylori* could induce proinflammatory gene expression* via* interaction with four of them, that is, TLR2, TLR4, TLR5, and TLR9 [1–4]. Expression of TLR2, TLR4, and TLR5 has also been detected in the gastric mucosa of* H. pylori* infected patients [1–4].

TLR signaling is mediated by two main pathways: MyD88 dependent (leading to the expression of proinflammatory cytokines) and MyD88 independent (responsible for interferon type I production). MyD88 is an adaptor protein that is used by all TLRs with the exception of TLR3, which utilizes exclusively the MyD88-independent pathway. TLR4 is unique, because it can induce both the MyD88-dependent and independent pathways [3, 5].

MyD88 expression in macrophages has been found to be essential for* H. pylori* induction of inflammatory cytokines (IL-6, IL-1*β*, IL-10, and IL-12) [2, 3, 5]. Both TLR2 and TLR4 proved to be crucial as signaling receptors for these responses in mouse macrophages [2, 5, 6]. MyD88 dependent signaling was also required for induction of protective immune responses (IL-17, antimicrobial peptides) against* H. felis *in animal models [6].

Another class of innate immunity molecules, which may be involved in the* H. pylori* mediated immune response, are triggering receptors expressed on myeloid cells (TREMs) [7]. TREM-1 is a 30-kDa glycoprotein of the Ig family which is expressed mainly on neutrophils and monocytes [7]. TREM-1 is engaged in amplification of TLR-dependent signals, as well as enhancement of NOD-like receptors (NLRs) mediated responses, including the NOD1 pathway involved in protection against* H. pylori* infection [8]. TREM-1 is also expressed in gastric mucosa epithelial cells, and its expression is elevated in the gastric mucosa of* H. pylori* infected adult patients [7]. TREM-2 is expressed mainly on macrophages and dendritic cells [9, 10]. Its activation results in induction of anti-inflammatory reactions [9, 11], but so far this receptor has not been studied in* H. pylori* infected patients.

CD163 is a cell-surface glycoprotein receptor that is highly expressed on most subsets of resident tissue macrophages [12]. The expression of CD163 is strongly induced by anti-inflammatory mediators, such as glucocorticoids and IL-10, and is inhibited by proinflammatory mediators such as IFN-*γ*, TNF-*α*, and others [13]. CD163 is a marker of anti-inflammatory M2 macrophages [14]. In contrast, M1 macrophages are associated with strong proinflammatory and cytotoxic responses induced by IFN-*γ*, TNF-*α*, and IL-6.* H. pylori* infected asymptomatic patients show mixed M1/M2 phenotype in their gastric mucosa [15]. M1 polarized macrophages can be identified by their contribution to high inflammatory responses, epithelial atrophy, and premalignant lesions, whereas CD163 plays a role in protective immunity against bacterial infection [16], so it may also be important in* H. pylori* infection.

The CD14 receptor is a cell surface molecule expressed on monocytes and macrophages and serves as a part of the LPS recognizing complex. Its presence is necessary for interaction with LPS and generation of signal transduction pathways leading to production of many proinflammatory cytokines. Interaction with LPS changes the CD14 expression [17]. However, interaction of* H. pylori* LPS with CD14 is rather weak, because of the structural features of* H. pylori* lipid A [17, 18]. Nevertheless, the expression level of CD14 may indicate an infiltration of the gastric mucosa by monocytes/macrophages, and it may change as a result of interaction with LPS.

The aim of this study was to examine the expression of innate immunity components (MyD88, TLR2, TLR4, CD14, TREM1, and TREM2) in relation to other mediators of the inflammation (IL-1*β*, IL-2, IL-6, IL-10, IL-12, TNF-*α*, and IFN-*γ*) in the gastric mucosa of* H. pylori* infected and uninfected children. The results were correlated with gastric inflammation scores and the density of* H. pylori* colonization.

## 2. Materials and Methods

### 2.1. Patients

The study was undertaken in accordance with the Helsinki declaration, with approval from the Ethics Committee of the Collegium Medicum at Nicolaus Copernicus University in Bydgoszcz, Poland. Informed consent was obtained from all the parents of patients and from patients older than 16 years.

Pediatric patients, from the Department of Pediatric Endoscopy and Gastrointestinal Function Testing, University Hospital in Bydgoszcz, Poland, displaying dyspeptic symptoms were eligible for inclusion. Exclusion criteria included (1) previous diagnosis of* H. pylori* infection and its treatment, (2) a history of antibiotic, antacid, H_2_ blocker, proton pump inhibitor, bismuth compound, or nonsteroidal anti-inflammatory drug use during the previous 4 weeks, (3) previous diagnosis of other inflammatory diseases, such as coeliac disease, inflammatory bowel disease, or allergy, and (4) gastric perforation or hemorrhage, history of abdominal surgery, or evidence of other gastrointestinal pathology.

Each subject underwent a urea breath test and endoscopic examination of the upper gastrointestinal tract. Three antral biopsies were taken from each patient. One biopsy was submerged in RNA*later* solution and frozen for real-time PCR analysis. The other specimens were formalin-fixed and embedded in paraffin, sectioned, and stained with hematoxylin and eosin for histological analysis. Biopsy specimens were graded for gastritis by two independent pathologists, according to the updated Sydney system.

A patient was considered* H. pylori* infected when the urea breath test and either the microscopic evaluation or the PCR analysis of the gastric mucosa were positive for* H. pylori*. A patient was considered not infected when all three tests were negative.

### 2.2. Molecular Methods

#### 2.2.1. Genotyping of* H. pylori* Obtained from Gastric Mucosa

The cagA status of* H. pylori* was determined by the PCR method, as described previously [19].

#### 2.2.2. Expression of IL-1*β*, IL-2, IL-6, IL-10, IL-12, IFN-*γ*, TNF-*α*, TLR2, TLR4, TREM1, TREM2, MyD88, CD14, and CD163 mRNA in Gastric Mucosa

RNA was isolated from gastric mucosa by using GenElute Mammalian Total RNA Kit (Sigma Aldrich, St. Luis, MO), according to the manufacturer's instructions. Isolated RNA was subjected to DNase digestion to remove DNA traces. Synthesis of cDNA was performed using the TaqMan Reverse Transcription Reagents kit (Applied Biosystems) and mixed primers (hexamers). The expression of mRNA of IL-1*β*, IL-2, IL-6, IL-10, IL-12, IFN-*γ*, TNF-*α*, TLR2, TLR4, TREM1, TREM2, MyD88, CD14, CD163, and the reference gene (G3PDH) were assessed by real-time PCR using primers published elsewhere [10, 20–23]. The amplification reaction was conducted in a volume of 25 *μ*L, using 1 *μ*L of cDNA, 12.5 *μ*L SYBR GreenPCR Master mix (Applied Biosys), and 250 nM of each primer in the thermocycler 7500 Real Time PCR System. All samples were run in duplicates and template negative controls were included in each run. The following reaction conditions were used: 95°C for 10 min (initial denaturation), followed by 40 cycles by denaturizing at 95°C for 15 sec and a 1 min extension at 60°C. Melting curve analysis was performed after each run, to control for product amplification and to ensure that no dimers interfered with the reaction. Cycle threshold (Ct) values were determined by SDS 1.2 software (Applied Biosystems). The expression levels of genes studied were calculated by the 2_−ΔΔCt_ method and results were expressed as relative values (fold change) in relation to the control group of* H. pylori*-negative or* cagA*-negative samples, after normalization to the expression levels of the endogenous control (G3PDH gene).

### 2.3. Expression of TNF-*α* and IFN-*γ* in Gastric Mucosa

Immunohistochemical staining of antral gastric mucosa biopsies was performed using primary mouse antibodies against TNF-*α* (1 : 100) and INF-*γ* (1 : 200) (Santa Cruz Biotechnology, Santa Cruz, CA, USA). Tissue sections were incubated with primary antibodies overnight at 4°C. The antigen-antibody complex was detected using Anti-Mouse EnVision HRP-Labeled Polymer (DakoCytomation, Glostrup, Denmark), a peroxidase detection system, and localized with DAB (3-3′diaminobenzidine) as a chromogen. Finally, the sections were counterstained with hematoxylin, dehydrated in increasing grades of ethyl alcohol (80, 90, 96, 99.8%), and mounted with Shandon Consul Mount (Thermo Scientific).

The expression of TNF-*α* and INF-*γ* was evaluated in epithelial cells and lamina propria of antral gastric mucosa, by using a light microscope, ECLIPSE E800 (Nikon Instruments Europe, Amsterdam, Netherlands).

The immunohistochemical expression of analyzed proteins was estimated as a percentage of positive cells multiplied by the intensity of staining, according to morphometric principles based on the modified Remmele-Stegner scale (IRS—Index Remmele-Stegner) [24]. The morphologic studies were performed at 20x original objective magnification. The final level of estimated protein expression was evaluated as the ratio of the expression intensity and the positively expressed number of cells/tissue area (total scale range 0–9). The number of positive immunoreactive area was categorized as 0, negative; 1 = 1 − 5 positive cells; 2 = 6 − 20 positive cells; 3 = ≥ 20 of positive cells. The intensity of staining was scored as follows: 0—negative, 1—low, 2—moderate, 3—strong.

During immunohistochemical staining, for determination of the appropriate antibody dilution, and elimination of false positive results, as well as for the reduction of the background reaction, a series of positive control reactions were performed on a model tissue selected according to the antibodies datasheet, and reference sources (The Human Protein Atlas http://www.proteinatlas.org). The positive control for TNF-*α* was performed on the kidney sections, and the representative expression was estimated in cells in tubules. The positive control for INF-*γ* was performed on the placenta, and the expression was estimated in trophoblastic cells. Moreover, negative immunohistochemical control reactions were performed, by substituting the primary antibody by a solution of diluted 1% BSA (bovine serum albumin) in PBS (phosphate buffered saline).

### 2.4. Statistical Analysis

Data were analyzed by the nonparametric Mann-Whitney* U* test. The relationship between histological parameters and the level of gene expression was evaluated using Spearman's correlation coefficient. Statistical calculations were made using STATISTICA 6.0 for Windows PL, with the level of statistical significance at *P* < 0.05.

## 3. Results

A total of 78 children were included in the study (55 girls; age range 7–18 years, mean 14.0). Infection of* H. pylori* was confirmed in 40 (51%) patients, 20 (50%) of whom were carriers of* cagA*-positive strains. None of the patients had peptic or duodenal ulcers.

### 3.1. Expression of Inflammatory Mediators in the Gastric Mucosa

**Figure 1:**
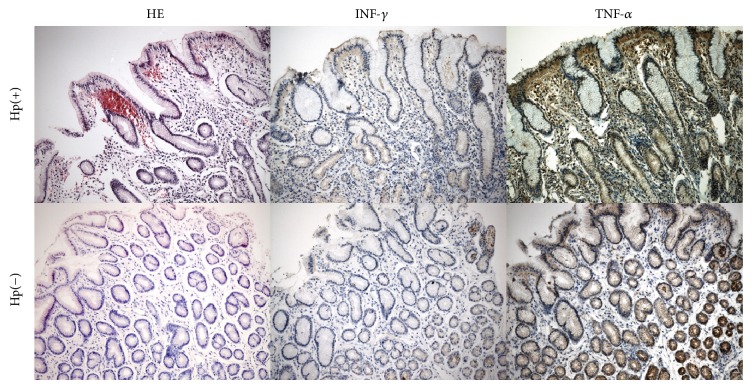
Hematoxylin-eosin (HE) staining and immunohistochemical staining with anti-IFN-*γ* and anti-TNF-*α* in* H. pylori* infected (Hp(+)) and noninfected children (Hp(−)). Magnification, ×20.

Significantly higher (from 3.4- to 6.5-fold) expression of* TNF-α*,* INF-γ*,* IL-6*,* IL-10,* and* CD163* mRNA was found in the gastric mucosa of* H. pylori* infected patients as compared to uninfected individuals (all *P* values <0.01). In contrast, mRNA expression of* TLR2*,* TLR4*,* TREM1*,* TREM2, CD14,* and* MyD88* did not differ between the two groups (Table 1). Higher expression of TNF-*α* and INF-*γ* in the gastric mucosa in* H. pylori*-positive patients was also detected by immunochemistry (*P* = 0.02; *P* < 0.01, Figure 1). INF-*γ* expression correlated with both the density of* H. pylori* colonization and lymphocytic infiltration in the gastric mucosa (*r* = 0.41, *P* < 0.001; *r* = 0.42, *P* < 0.01), whereas TNF-*α* expression correlated only with bacterial density (*r* = 0.51, *P* = 0.02). No correlation between expression of the remaining immunological markers and the intensity of inflammation or bacterial load in the gastric mucosa was noted.

Children infected with the* cagA*-positive strain had higher levels of IL-6 (2.5-fold, *P* = 0.03) and IL-10 (3.5-fold, *P* < 0.01) mRNA than those with the cagA-negative strain, whereas no differences were found for other markers studied (Table 1).

## 4. Discussion

This study showed that* H. pylori* infection in children resulted in mRNA up-regulation of* IL-6*,* IL-10*,* TNF-α*,* IFN-γ*, and* CD163* and unchanged expression of* MyD88*,* TLR2*, and* TLR4* mRNA in the gastric mucosa.* H. pylori cagA*(+) infection was connected with an upregulation of* IL-10* and* IL-6* mRNA expression. These data confirm the results of other studies showing the Th1 profile of* H. pylori*-mediated inflammation [25, 26]. However, these changes occurred without induction of basic TLRs system components (MyD88, TLR2, and TLR4) and other innate immunity molecules (TREM1, TREM2, and CD14) [2, 4, 26, 27].

One of the key molecules is MyD88 intracellular adaptor protein, which is necessary for mediating signals from all TLRs except TLR3 [3]. MyD88 dependent signaling pathways are involved in induction of several inflammatory cytokines (IL-6, IL-1*β*, IL-12, and IL-10) in the bone marrow-derived macrophages, which enables the elimination of the pathogen and protects against tissue damage [2, 5, 28].

Our results suggest that* H. pylori *may modulate MyD88 expression. Lack of significant MyD88 induction by* H. pylori* may be responsible for infection persistence and induction of endotoxin tolerance, which may lead to a reduced inflammatory response after repeated challenge by LPS [28, 29].

In contrast to Enterobacteriaceae, LPS of* H. pylori *is less immunogenic and does not use the TLR4 pathway but induces mainly TLR2-mediated signaling [6, 30–32]. The same pathway is used by* P. gingivalis *LPS. It uses TLR-2 mediated activation signaling that is associated with impaired endotoxin tolerance, neutrophil-dominated chronic inflammation, elevated levels of IL-8 and MIP-2, but low production of IFN-*β* [18, 23]. It is unclear whether these observations can be applied to* H. pylori*-mediated inflammation.

The described lack of or poor TLR4 engagement in* H. pylori* recognition may be at least partially explained by unchanged gastric mucosa* CD14* mRNA expression level. The main CD14 function is its interaction with LPS and induction of TLR4/MD2 mediated signaling pathway engaged in activation of many proinflammatory reactions [33]. So the unchanged CD14 expression found here may result in its impaired engagement in* H. pylori*-LPS-mediated mucosal inflammation [34, 35].

These data might suggest that gastric mucosa* IL-6*,* TNF-α*,* IL-10,* and* CD163 *mRNA upregulation found here possibly did not depend on LPS-mediated signaling because* H. pylori* infection did not change CD14 transcript levels. Gastric mucosa* CD14* mRNA expression depends on the level of mucosal infiltration by macrophages and neutrophils, and their activation status [33].* H. pylori* infected adults showed increased CD14 expression in the gastric mucosa, especially in gastric tumor tissues [36].

On the other hand, our results contrast those of* H. pylori* infected adults, who showed an increased expression of TLR2 and TLR4 in the gastric mucosa [37]. Also, a recent study in a group of 50 children from Mexico City showed that* H. pylori* infection was associated with increased expression of TLR2, TLR4, TLR5, and TLR9 proteins in the gastric epithelium, as well as up-regulation of the cytokines IL-10, IL-8, and TNF-*α* [26]. These discrepancies may stem from ethnic characteristics or children involved in both studies. The Mexican group consisted mostly of the Mexican population, which expressed Amerindian genetic markers. These divergent results may also result from differences in the pathogenicity of infecting* Helicobacter pylori* strains and TLRs genetic polymorphisms [38].

We previously found that* H. pylori* infection in children is associated with systemic activation of circulating monocytes (upregulation of CD11b, CD11c, and CD18), which is downregulated following eradication therapy [39]. This observation is partially consistent with the elevation of CD163 and IL-10 mRNA expression in the gastric mucosa of* H. pylori*-infected children. CD163 is a cell surface molecule that is expressed exclusively on resident tissue macrophages [12], therefore high CD163 mRNA in the gastric mucosa of infected children may be associated with increased numbers of activated peripheral blood monocytes migrating into sites of inflammation in the gastric mucosa, where they may eventually turn into macrophages with high CD163 expression.

The role of CD163 in* H. pylori* infection is not known. As this molecule binds both Gram-positive and Gram-negative bacteria, it may contribute to the host defense against infection [13, 16]. On the other hand, CD163 is widely recognized as a marker of M2 macrophages [13, 14, 22, 40]. M2 macrophages exhibit anti-inflammatory and immune-modulating functions and induce mainly Th2 responses [16], which do not contribute to* H. pylori* elimination. Depending on the induction agent, M2 macrophages can be divided into at least three different subpopulations, with high IL-10 synthesis as a common feature. High expression of* IL-10* mRNA in the gastric mucosa, documented in this study, may, at least partially, originate from M2 macrophages, which are highly increased in the gastric mucosa of* H. pylori* infected subjects [15, 34]. Additionally, IL-10 upregulates expression of* CD163 *and other monocyte anti-inflammatory genes like* IL-1 receptor antagonist* (*IL-1r*) [41] or* suppressors of cytokine signaling-3* (*SOCs-3*) [42], which downregulate immune responses. These findings confirm recent data indicating that* H. pylori*-mediated inflammation is related to the generation of tolerogenic macrophages and dendritic cells contributing to the formation of different types of suppressor T cells (Treg, Tr1, and Th3). The latter are especially numerous and active in children [43, 44] and in a mouse model system [45].

We found that the gastric expression of TREM 1 and TREM 2 mRNA was not affected by* H. pylori* infection [7]. In contrast,* H. pylori* infected adults had elevated expression of TREM1 in gastric epithelial cells. TREM1 expression in the gastric mucosa also reflects the extent of macrophages and neutrophils infiltration. TREM 2 acts antagonistically to TREM 1 and promotes anti-inflammatory response [9]. It can also negatively affect TLR-dependent response [11]. Lack of changes in TREM1 expression found in our study further confirms the tolerogenic status of leucocytes present in the gastric mucosa of* H. pylori*-infected children, since their activation leads to TREM 1 and TREM 2 upregulation [7–11]. Together, these results suggest that the TLRs system is poorly involved in* H. pylori*-induced inflammation in the gastric mucosa in children, and that other PRRs may be more engaged in pathogen recognition [3].

The positive correlation between the lymphocyte infiltration and IFN-*γ* found in this study indicates that lymphocytes (T and NK cells) infiltrating the gastric mucosa may produce IFN-*γ* and switch the response towards a Th1 profile, as has previously been described in other studies [3, 25, 30, 35]. Th1 response did not limit* H. pylori* colonization, because its load correlated with TNF-*α* and INF-*γ* expression in the gastric mucosa. These observations confirm previous studies in adults and children, showing that Th1 response is not protective [30].

## 5. Conclusions

To summarize, this study demonstrated that* H. pylori* infection in children was characterized by (a) nonprotective Th1 response associated with high* H. pylori* load and increased lymphocytic infiltration into gastric mucosa and (b) presence of anti-inflammatory activities (high expression of CD163 and IL-10 mRNA). All of these activities were induced without significant activation of innate immunity components, such as TLRs system molecules (TLR2, TLR4, and MyD88), and inflammatory markers of macrophages (TREM1, TREM2). The ability of* H. pylori* to manipulate the immune response (activation or inactivation of TLR-dependent response) may be responsible for bacterial survival and a mild course of infection in children.

## Figures and Tables

**Table 1:** mRNA expression of inflammatory mediators in the gastric mucosa in relation to *H. pylori* and *cagA* status.

Gene	Expression in *H. pylori* infected relative to noninfected patients	*P* value	Expression in *H. pylori cagA*(+) relative to *H. pylori cagA*(−) patients	*P* value
IL-1*β*	1.1	*0.06 *	1.5	*0.13 *
IL-2	1.4	*0.86 *	1.4	*0.29 *
IL-6	**4.6**	***<0.01***	**2.5**	***0.03***
IL-10	**6.5**	***<0.01***	**3.5**	***<0.01***
IL-12*β*	1.2	*0.56 *	1.0	*0.95 *
INF-*γ*	**3.4**	***<0.01***	1.6	*0.26 *
TNF-*α*	**5.5**	***<0.01***	1.0	*0.25 *
MyD88	1.4	*0.54 *	0.8	*0.67 *
TLR2	1.7	*0.30 *	1.2	*0.58 *
TLR4	1.5	*0.11 *	1.2	*0.48 *
TREM1	1.4	*0.39 *	1.1	*0.4 *
TREM2	1.4	*0.23 *	1.2	*0.31 *
CD14	1.5	*0.43 *	1.0	*0.99 *
CD163	**4.2**	***<0.01***	1.4	*0.44 *

The expression of each gene is given relative to the expression in *H. pylori*-negative or *H. pylori  cagA*-negative samples (fold change in log_10_ RQ).

## References

[1] Pimentel-Nunes P., Afonso L., Lopes P. (2011). Increased expression of toll-like receptors (TLR) 2, 4 and 5 in gastric dysplasia. *Pathology and Oncology Research*.

[2] Rad R., Brenner L., Krug A. (2007). Toll-like receptor-dependent activation of antigen-presenting cells affects adaptive immunity to *Helicobacter pylori*. *Gastroenterology*.

[3] Müller A., Oertli M., Arnold I. C. (2011). *H. Pylori* exploits and manipulates innate and adaptive immune cell signaling pathways to establish persistent infection. *Cell Communication and Signaling*.

[4] Schmaußer B., Andrulis M., Endrich S. (2004). Expression and subcellular distribution of toll-like receptors TLR4, TLR5 and TLR9 on the gastric epithelium in Helicobacter pylori infection. *Clinical and Experimental Immunology*.

[5] Obonyo M., Sabet M., Cole S. P. (2007). Deficiencies of myeloid differentiation factor 88, toll-like receptor 2 (TLR2), or TLR4 produce specific defects in macrophage cytokine secretion induced by *Helicobacter pylori*. *Infection and Immunity*.

[6] Mandell L., Moran A. P., Cocchiarella A. (2004). Intact gram-negative *Helicobacter pylori*, *Helicobacter felis*, and *Helicobacter hepaticus* bacteria activate innate immunity via toll-like receptor 2 but not toll-like receptor 4. *Infection and Immunity*.

[7] Schmaußer B., Endrich S., Beier D. (2008). Triggering receptor expressed on myeloid cells-1 (TREM-1) expression on gastric epithelium: implication for a role of TREM-1 in *Helicobacter pylori* infection. *Clinical and Experimental Immunology*.

[8] Netea M. G., Azam T., Ferwerda G., Girardin S. E., Kim S.-H., Dinarello C. A. (2006). Triggering receptor expressed on myeloid cells-1 (TREM-1) amplifies the signals induced by the NACHT-LRR (NLR) pattern recognition receptors. *Journal of Leukocyte Biology*.

[9] Sun G.-Y., Guan C.-X., Zhou Y. (2011). Vasoactive intestinal peptide re-balances TREM-1/TREM-2 ratio in acute lung injury. *Regulatory Peptides*.

[10] Radhakrishnan S., Arneson L. N., Upshaw J. L. (2008). TREM-2 mediated signaling induces antigen uptake and retention in mature myeloid dendritic cells. *The Journal of Immunology*.

[11] Ito H., Hamerman J. A. (2012). TREM-2, triggering receptor expressed on myeloid cell-2, negatively regulates TLR responses in dendritic cells. *European Journal of Immunology*.

[12] van den Heuvel M. M., Tensen C. P., van As J. H. (1999). Regulation of CD163 on human macrophages: cross-linking of CD163 induces signaling and activation. *Journal of Leukocyte Biology*.

[13] Ritter M., Buechler C., Langmann T., Orso E., Klucken J., Schmitz G. (2000). The scavenger receptor CD163: regulation, promoter structure and genomic organization. *Pathobiology*.

[14] Komohara Y., Hirahara J., Horikawa T. (2006). AM-3K, an anti-macrophage antibody, recognizes CD163, a molecule associated with an anti-inflammatory macrophage phenotype. *Journal of Histochemistry and Cytochemistry*.

[15] Quiding-Jarbrink M., Raghavan S., Sundquist M. (2010). Enhanced M1 macrophage polarization in human *Helicobacter pylori*-associated atrophic gastritis and in vaccinated mice. *PLoS ONE*.

[16] Fabriek B. O., Bruggen R. V., Deng D. M. (2009). The macrophage scavenger receptor CD163 functions as an innate immune sensor for bacteria. *Blood*.

[17] Bliss C. M., Golenbock D. T., Keates S., Linevsky J. K., Kelly C. P. (1998). *Helicobacter pylori* lipopolysaccharide binds to CD14 and stimulates release of interleukin-8, epithelial neutrophil-activating peptide 78, and monocyte chemotactic protein 1 by human monocytes. *Infection and Immunity*.

[18] Cunningham M. D., Seachord C., Ratcliffe K., Bainbridge B., Aruffo A., Darveau R. P. (1996). *Helicobacter pylori* and *Porphyromonas gingivalis* lipopolysaccharides are poorly transferred to recombinant soluble CD14. *Infection and Immunity*.

[19] Gzyl A., Dzierzanowska D., Rozynek E., Celińska-Cedro D., Dura W., Berg D. E. (1999). PCR-based diagnosis of *Helicobacter pylori* infection in Polish children and adults. *Journal of Medical Microbiology*.

[20] Galal N., El-Beialy W., Deyama Y. (2010). Up-regulation of the G3PDH “housekeeping” gene by estrogen. *Molecular Medicine Reports*.

[21] Ornatowska M., Azim A. C., Wang X. (2007). Functional genomics of silencing TREM-1 on TLR4 signaling in macrophages. *American Journal of Physiology—Lung Cellular and Molecular Physiology*.

[22] Schaer C. A., Vallelian F., Imhof A., Schoedon G., Schaer D. J. (2007). CD163-expressing monocytes constitute an endotoxin-sensitive Hb clearance compartment within the vascular system. *Journal of Leukocyte Biology*.

[23] Gangloff M., Gay N. J. (2004). MD-2: the Toll “gatekeeper” in endotoxin signalling. *Trends in Biochemical Sciences*.

[24] Remmele W., Stegner H. E. (1987). Recommendation for uniform definition of an immunoreactive score (IRS) for immunohistochemical estrogen receptor detection (ER-ICA) in breast cancer tissue. *Pathologe*.

[25] Luzza F., Parrello T., Sebkova L. (2001). Expression of proinflammatory and Th1 but not Th2 cytokines is enhanced in gastric mucosa of *Helicobacter pylori* infected children. *Digestive and Liver Disease*.

[26] Lagunes-Servin H., Torres J., Maldonado-Bernal C. (2013). Toll-like receptors and cytokines are upregulated during *Helicobacter pylori* infection in children. *Helicobacter*.

[27] Rad R., Ballhorn W., Voland P. (2009). Extracellular and intracellular pattern recognition receptors cooperate in the recognition of *Helicobacter pylori*. *Gastroenterology*.

[28] Sato S., Takeuchi O., Fujita T., Tomizawa H., Takeda K., Akira S. (2002). A variety of microbial components induce tolerance to lipopolysaccharide by differentially affecting MyD88-dependent and -independent pathways. *International Immunology*.

[29] Zaric S. S., Coulter W. A., Shelburne C. E. (2011). Altered toll-like receptor 2-mediated endotoxin tolerance is related to diminished interferon *β* production. *The Journal of Biological Chemistry*.

[30] Amedei A., Cappon A., Codolo G. (2006). The neutrophil-activating protein of *Helicobacter pylori* promotes Th1 immune responses. *Journal of Clinical Investigation*.

[31] Erridge C., Pridmore A., Eley A., Stewart J., Poxton I. R. (2004). Lipopolysaccharides of *Bacteroides fragilis*, *Chlamydia trachomatis* and *Pseudomonas aeruginosa* signal via Toll-like receptor 2. *Journal of Medical Microbiology*.

[32] Yokota S.-I., Ohnishi T., Muroi M., Tanamoto K.-I., Fujii N., Amano K.-I. (2007). Highly-purified *Helicobacter pylori* LPS preparations induce weak inflammatory reactions and utilize Toll-like receptor 2 complex but not Toll-like receptor 4 complex. *FEMS Immunology and Medical Microbiology*.

[33] Dzierzanowska-Fangrat K., Michalkiewicz J., Cielecka-Kuszyk J. (2008). Enhanced gastric IL-18 mRNA expression in Helicobacter pylori-infected children is associated with macrophage infiltration, IL-8, and IL-1*β* mRNA expression. *European Journal of Gastroenterology and Hepatology*.

[34] Fehlings M., Drobbe L., Moos V. (2012). Comparative analysis of the interaction of *Helicobacter pylori* with human dendritic cells, macrophages, and monocytes. *Infection and Immunity*.

[35] Yun C. H., Lundgren A., Azem J. (2005). Natural killer cells and *Helicobacter pylori* infection: bacterial antigens and interleukin-12 act synergistically to induce gamma interferon production. *Infection and Immunity*.

[36] Shimazu R., Akashi S., Ogata H. (1999). MD-2, a molecule that confers lipopolysaccharide responsiveness on toll- like receptor 4. *Journal of Experimental Medicine*.

[37] Asahi K., Hai Y. F., Hayashi Y. (2007). *Helicobacter pylori* infection affects toll-like receptor 4 expression in human gastric mucosa. *Hepato-Gastroenterology*.

[38] Pimentel-Nunes P., Teixeira A. L., Pereira C. (2013). Functional polymorphisms of Toll-like receptors 2 and 4 alter the risk for colorectal carcinoma in Europeans. *Digestive and Liver Disease*.

[39] Helmin-Basa A., Czerwionka-Szaflarska M., Bala G. (2012). Expression of adhesion and activation molecules on circulating monocytes in children with *Helicobacter pylori* infection. *Helicobacter*.

[40] Buechler C., Ritter M., Orsó E., Langmann T., Klucken J., Schmitz G. (2000). Regulation of scavenger receptor CD163 expression in human monocytes and macrophages by pro- and antiinflammatory stimuli. *Journal of Leukocyte Biology*.

[41] Joyce D. A., Gibbons D. P., Green P., Steer J. H., Feldmann M., Brennan F. M. (1994). Two inhibitors of pro-inflammatory cytokine release, interleukin-10 and interleukin-4, have contrasting effects on release of soluble p75 tumor necrosis factor receptor by cultured monocytes. *European Journal of Immunology*.

[42] Donnelly R. P., Dickensheets H., Finbloom D. S. (1999). The interleukin-10 signal transduction pathway and regulation of gene expression in mononuclear phagocytes. *Journal of Interferon and Cytokine Research*.

[43] Freire de Melo F., Rocha A. M. C., Rocha G. A. (2012). A regulatory instead of an IL-17 T response predominates in *Helicobacter pylori*-associated gastritis in children. *Microbes and Infection*.

[44] Gil J. H., Seo J. W., Cho M.-S., Ahn J.-H., Sung H. Y. (2014). Role of Treg and TH17 cells of the gastric mucosa in children with *Helicobacter pylori* gastritis. *Journal of Pediatric Gastroenterology and Nutrition*.

[45] Laurie K. L., van Driel I. R., Gleeson P. A. (2002). The role of CD4^+^CD25^+^ immunoregulatory T cells in the induction of autoimmune gastritis. *Immunology and Cell Biology*.

